# A novel case of compound heterozygous congenital hyperinsulinism without high insulin levels

**DOI:** 10.1186/s13633-015-0012-4

**Published:** 2015-07-15

**Authors:** Cassandra Brady, Andrew A. Palladino, Iris Gutmark-Little

**Affiliations:** Cincinnati Children’s Hospital Medical Center, Division of Endocrinology, 3333 Burnet Ave, MLC 7012, Cincinnati, OH 45229 USA; Children’s Hospital of Philadelphia, Division of Endocrinology, 34th Street and Civic Center Boulevard, Philadelphia, PA 19104 USA

**Keywords:** Congenital hyperinsulinism, *ABCC8*, Octreotide, Diazoxide-unresponsive

## Abstract

**Background:**

Congenital hyperinsulinism leads to unregulated insulin secretion and hypoglycemia. Diagnosis can be difficult and genetic testing may be warranted.

**Case:**

This patient initially presented at 11 months with seizure activity secondary to severe hypoglycemia. Her diagnostic evaluation included genetic studies, which confirmed congenital hyperinsulinism. A novel combination of mutations in the *ABCC8* gene leading to diffuse, diazoxide-unresponsive congenital hyperinsulinism was identified. Mutation analysis of *ABCC8* showed three variants (R1215W – paternal, pathogenic; W739C – maternal, variant of unknown significance; R1393L – maternal, variant of unknown significance). Her clinical course continues to be complicated by severe, refractory hypoglycemia at age 3 years.

**Conclusion:**

We describe a novel compound heterozygous mutation leading to diffuse, diazoxide-unresponsive congenital hyperinsulinism. This case illustrates challenges associated with diagnosing and managing congenital hyperinsulinism and the importance of genetic testing.

## Background

Congenital hyperinsulinism (CHI) leads to hypoglycemia in infants and children due to inappropriately elevated insulin levels. Mutations leading to CHI have been described in nine genes [1]. The most common mutations occur in *ABCC8* and *KCNJ11*, which encode for the ATP-sensitive potassium (K_ATP_) channel present on the beta-cell plasma membrane. During normal glucose-stimulated insulin secretion, the K_ATP_ channel closes in response to an increase in the energy potential of the beta-cell. Closure of this channel results in depolarization of the beta-cell plasma membrane leading to calcium influx into the beta-cell and insulin secretion. Certain mutations in *ABCC8* and *KCNJ11* lead to defective K_ATP_ channels that are either unable to open or cannot localize to the plasma membrane [2]. Defective channels lead to continued depolarization of the plasma membrane and insulin secretion regardless of the plasma glucose level. Patients with these mutations can be classified as diazoxide-responsive or unresponsive [1, 3]. Diazoxide is a K_ATP_ channel agonist which keeps the channel in an open state resulting in the suppression of insulin secretion [4]. Severe forms of CHI can lead to complete destruction of channel activity and are therefore unresponsive to diazoxide [2]. Inheritance of *ABCC8* and *KCNJ11* mutations occurs in either a dominant or recessive fashion [1]. These mutations, dependent on inheritance pattern, may lead to focal or diffuse disease histologically (i.e. localized islet cell abnormalities versus effects throughout pancreas). Homozygous or compound heterozygous recessive mutations are associated with diffuse disease, and are typically managed medically or with near–total pancreatectomy, as indicated.

The diagnosis of CHI can be made during a fasting challenge. At the time of hypoglycemia (glucose <50 mg/dL (2.775 mmol/L)) [2, 3], a blood sample (“critical sample”) is obtained to assess the presence or absence of key metabolic indices and counter-regulatory hormones which serve as alternative fuels and stimulants to raise serum glucose. Often, the fasting challenge does not yield confirmatory or clear diagnostic evidence of CHI and a repeat fasting evaluation may be necessary. Genetic testing is also recommended to assess for the common CHI-causing mutations in patients with a known or suspected diagnosis of hyperinsulinism.

Once CHI is confirmed, defining the extent of disease is a critical next step to determine ideal intervention. Previously, imaging studies such as ultrasound, computed tomography, and magnetic resonance imaging have been implemented for the purpose of distinguishing focal from diffuse disease. However, these studies have not been shown to be effective for this purpose. The radiotracer ^18^fluoro-L- dihydroxyphenylalanine (^18^ F-DOPA), which concentrates in insulin-secreting beta cells of the pancreas, has been used in specialized CHI care centers for the past decade to classify disease. In the largest series, the specificity of this imaging technique to detect focal disease was 96 % [5].

This case demonstrates a novel compound heterozygous mutation causing CHI, the natural history of this mutation, and the associated challenges in management. This case also outlines the pitfalls of interpreting insulin levels at the time of hypoglycemia and emphasizes the importance of genetic testing in this setting.

## Case presentation

An 11-month-old African-American female presented to neurology due to seizure-like activity. She was a former term infant with a birthweight of 3.714 kg. At presentation she weighed 9.6 kg (56th%) and was 77 cm (89th%) tall. During her evaluation, a glucose value of 38 mg/dL (1.998 mmol/L) and bicarbonate of 24 mmol/L were noted.

During a subsequent fasting challenge, her serum glucose decreased to 32 mg/dL (1.776 mmol/L) within 5.5 h. The results of the critical sample are presented in Table 1. Specifically, her insulin level was appropriately low, while her beta-hydroxybutyrate (BOHB) was minimally elevated, and free fatty acids were inappropriately within the normal range. Additionally, her growth hormone was low. All other laboratory results including ammonia, *urine organic acids, *serum amino acids, and *acylcarnitine profile were normal (*drawn in the fed state). A glucagon stimulation to assess glycemic response at the time of hypoglycemia was attempted but was unsuccessful. Based on the critical sample results, she was thought to have growth hormone deficiency and was started on growth hormone therapy. She was discharged after several inpatient days with improved glucose levels.

**Table 1:** Fasting challenge results

Test name (Normal range)	Laboratory result
IGFBP-1 (ng/mL) (5–9 years: 15–95; no reference range for patients <5 years)	61
Insulin (<2-13 μIU/mL fasting)	0.2
Glucose (>70 mg/dL/3.885 mmol/L)	32/1.776
C-peptide (0.8-3.5 ng/mL)	0.5
BOHB (0–3.0 mg/dL)	4.3 (0.41 mmol/L)
Free Fatty Acids (0.5-0.9 mmol/L)	0.46
Growth Hormone (>7 ng/mL)	3

In the interim, the insulin-like growth factor-1 (IGF-1) (prior to growth hormone therapy) resulted at 105.7 ng/mL (approximately the 80th% for age and sex), and insulin-like growth factor binding protein-3 (IGFBP-3) was 2800 ng/mL (above the mean for age and sex). While at home, she was noted to have a glucose of 19 mg/dL (1.0545 mmol/L) despite growth hormone therapy. Given the severity of her hypoglycemia, she was readmitted for further assessment. An arginine/clonidine growth hormone stimulation test done after discontinuation of growth hormone revealed a peak growth hormone level of 7.9 ng/dL (radioimmunoassay). In the setting of continued hypoglycemia despite growth hormone therapy, her critical sample obtained at her initial admission was reviewed and determined to be concerning for CHI. Therefore genetic studies for CHI were sent (Athena Diagnostics, Worcester, Massachusetts).

While genetic studies were pending, diazoxide treatment was initiated and titrated to 15 mg/kg/day divided three times daily. However, she continued to have hypoglycemia. After one week of diazoxide therapy, octreotide was added. Octreotide was increased to 15 mcg/kg/day, with subsequent improvement but not resolution of hypoglycemia. Frequent feeds and uncooked cornstarch were implemented to avert hypoglycemia. Her hyperinsulinism was deemed diazoxide-unresponsive and diazoxide was discontinued.

Mutational analysis confirmed a diagnosis of CHI as three variants in *ABCC8* were identified (Table 2). The R1215W variant was found to be paternally-inherited while the W739C and R1393L variants were maternally inherited. The paternal change was a known mutation described previously (at Children’s Hospital of Philadelphia) in a patient with diffuse disease. In this previously described patient, the inheritance pattern was thought to be recessive, given a normal maternal *ABCC8* gene copy. In our patient, the significance of the maternal variants was unclear as they had not been previously described in CHI cases. Therefore, for our patient, we were unable to predict focal versus diffuse disease pathology based on mutational analysis alone.

**Table 2:** Genetic results (*ABCC8 Mutation)*

Mutation	Inheritance	Interpretation
c. R1215W	Paternal	Pathogenic
c. W739C	Maternal	VUS
c. R1393L	Maternal	VUS

*VUS* variant of unknown significance

**Fig. 1:**
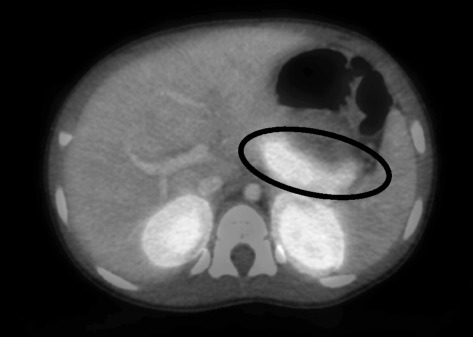
Results of ^F^-Dopa PET Scan. This image demonstrates the results of our patient’s ^F^-Dopa PET scan. The pancreas is circled in black. There is no evidence of focal tracer uptake on this image, suggesting diffuse disease. However the finding of diffuse uptake on ^F^-Dopa PET scan does not exclude a focal lesion

Our patient was transferred to Children’s Hospital of Philadelphia for an 18-fluoro L-3, 4-dihydroxyphenylalanine positron emission tomography (^18^-F-DOPA-PET) scan. There was no focal uptake of isotope by the pancreas (Fig. 1), which suggested, but did not confirm diffuse disease. A pancreatic biopsy was subsequently recommended in order to further delineate her pathology, but was declined by the family. A gastrostomy tube was placed for enteral dextrose administration (overnight glucose infusion rate (GIR) of 7 mg/kg/min).

Over several months, the child has continued to have intermittent, daily hypoglycemia despite adjustments of her treatment regimen. The GIR of her fluids has been gradually increased. Her current treatment regimen at age 3 years consists of octreotide 20 mcg/kg/day divided three times daily and overnight dextrose (14 h, GIR of 9 mg/kg/min) through her gastrostomy tube. She continues to have daytime hypoglycemia [glucose <70 mg/dL (3.885 mmol/L)]. Speech delay that was noted shortly after diagnosis persists despite therapy. Considering her disease and failure of medical management, a near-total pancreatectomy may be necessary to achieve normal blood glucose levels. Other treatment options that are also being considered include continuous glucagon via an insulin pump [6, 7] and continuous octreotide via an insulin pump [8]. These treatments have not been pursued based on parental requests.

## Conclusions

This case reveals a new compound heterozygous mutation in *ABCC8* that leads to diffuse, diazoxide-unresponsive CHI. It highlights the severity of disease due to this combination of mutations and the difficulty of diagnosis and management.

To diagnose CHI, laboratory tests known as the “critical sample”, are utilized and include: plasma glucose, plasma insulin, c-peptide, insulin-like growth factor binding protein-1 (IGFBP-1), BOHB, free fatty acids, ammonia, lactate, pyruvate, acylcarnitine profile, free and total carnitine, organic acids (urine), growth hormone, and cortisol [3]. The diagnosis of CHI can be made in the setting of hypoglycemia when elevated insulin levels are present with suppression of BOHB and free fatty acids. Often, the initial fasting challenge does not yield confirmatory or clear diagnostic evidence of CHI and repeat fasting evaluations may be necessary.

In the critical sample, insulin and c-peptide levels help to establish the presence of inappropriate elevations in insulin and to distinguish between excess endogenous and exogenous insulin, respectively. Interpretation of insulin levels at the time of hypoglycemia can be difficult, as evidenced by this case. Insulin levels may be elevated, decreased, or normal [9]. This may be due to periodic release of insulin [3], insulin degrading enzymes in hemolyzed blood samples [9–11], insulin antibodies [12], and rapid hepatic clearance [9, 13]. At room temperature, research shows that >90 % of insulin can be degraded due to massive hemolysis which induces degrading enzymes [11]. Maintaining the sample at colder temperatures (4 °C) may decrease degrading enzyme activity [11], however this can make critical sample collection more difficult. Interfering insulin antibodies may also lead to falsely low insulin levels as demonstrated in immunoradiometric assays and electrochemiluminescent assays in prior reports [12]. Insulin is also rapidly degraded by the body at a rate of 2 percent per minute with about 50 % removed by the liver [13, 9]. These issues may have played a role in our patient’s decreased insulin level on her fasting challenge.

Another important indicator of hyperinsulinism is the glycemic response to glucagon at the time of hypoglycemia [14]. A glucose rise after glucagon of greater than 30 mg/dL (1.665 mmol/L) may reflect hyperinsulinism with a sensitivity of 91 % and specificity of 93 % [14, 9]. This glycemic excursion after glucagon reflects the suppressive effects of insulin on the release of glycogen stores from the liver. In our patient’s evaluation, the glucagon challenge was not completed in a way that it would have been interpretable.

The fasting challenge is an important opportunity to obtain and interpret other counter-regulatory mechanisms that maintain euglycemia. Elevation in BOHB is a measure of appropriate ketogenesis during fasting. BOHB values less than 2.7 mmol/liter (28.1 mg/dL) during hypoglycemia have a reported sensitivity and specificity of 100 % in hyperinsulinemia induced by an insulinoma [9, 15]. The patient in this case had a relatively low BOHB value, which may have been an indicator of high insulin levels. Growth hormone and cortisol act as counter-regulatory hormones to increase lipolysis and gluconeogenesis, respectively. Growth hormone and cortisol are expected to be increased during hypoglycemia if these hormonal systems are intact [16]. The child in this case was started on growth hormone due to the low value at the time of hypoglycemia. However, decreased growth hormone and cortisol levels at the time of hypoglycemia are not diagnostic of deficiencies, and 65 % of children in a study by Kelly et al. were misdiagnosed with growth hormone deficiency during a fasting challenge [17]. Our patient subsequently had a normal growth hormone response during further evaluation.

Shortly after diagnosis in our patient’s clinical course, it became evident that the CHI was likely diazoxide-unresponsive. Diazoxide-unresponsive CHI is typically due to defects in the K_ATP_ channel [18]. However genetic analysis and determination of inheritance pattern is extremely helpful for both prediction of disease pathology and response to therapy. Inheritance of *ABCC8* and *KCNJ11* mutations occurs in either a dominant or recessive fashion [1]. Further, depending on inheritance pattern, these mutations may lead to focal or diffuse disease histologically. Autosomal recessive mutations from both parents typically cause diffuse disease. A paternally-inherited recessive mutation in the K_ATP_ channel with maternal loss of heterozygosity leads to paternal uniparental disomy and the development of focal disease [3, 1]. The genetic results for this patient demonstrated a paternally-inherited known pathogenic recessive mutation, but the pathogenicity of the maternal mutations was unclear. We have recently learned (from a patient at Children’s Hospital of Philadelphia) that the W739C maternal variant has been identified in a patient with diffuse disease indicating that it likely acts recessively. We suspect the R1393L variant is pathogenic and could act either recessively or dominantly. Of note, our patient’s brother is a carrier of the paternal mutation, but did not inherit either of the maternal mutations. Both of her parents and her brother have no signs or symptoms of hypoglycemia.

An ^18^-F-Dopa-PET scan was obtained. This noninvasive imaging technique has been used to further delineate between focal and diffuse CHI [19]. However, diffuse uptake on this scan does not rule out focal disease [3]. In the largest series, the specificity of this imaging technique to detect focal disease was 96 %, with a positive predictive value of 96 %. In this series, 105 patients with confirmed CHI underwent pancreatectomy. Of these, 53 had a postoperative diagnosis of focal CHI. However, preoperative ^18^-F-Dopa-PET imaging in 8 of these 53 was reported erroneously as diffuse [5].

Finally, there are reports of clinical improvement in individuals with heterozygous *ABCC8* mutations [20] or those with severe disease [21] with time. Whether improvement will occur in this patient, remains to be determined, but is becoming less likely. We speculate that the late presentation of this patient is more likely due to late detection as opposed to the genetic mutations.

We describe a novel compound heterozygous mutation leading to diffuse, diazoxide-unresponsive CHI. This case illustrates the pitfalls and challenges associated with diagnosing CHI and the importance of genetic testing and how it can guide management decisions, including whether or not imaging studies are warranted. Lastly, the case illustrates the difficulty of medically-managing a patient with diffuse CHI.

## Consent

Written informed consent was obtained from the patient for publication of this case report and any accompanying images. A copy of the written consent is available for review by the Editor-in-Chief of this journal.

## Abbreviations

CHI: Congenital hyperinsulinism
K_ATP_: ATP-sensitive potassium
IGF-1: Insulin-like growth factor-1
IGFBP-3: Insulin-like growth factor binding protein-3
IGFBP-1: Insulin-like growth factor binding protein-1
BOHB: beta-hydroxybutyrate
GIR: Glucose infusion rate
^18^-F-DOPA-PET: 18-fluoro L-3,4-dihydroxyphenylalanine positron emission tomography
^18^-F-DOPA: 18-fluoro L-3,4-dihydroxyphenylalanine
